# Low copper levels measured in the aortic wall of New Zealand patients with non-syndromic ascending thoracic aortic aneurysm

**DOI:** 10.1093/icvts/ivac235

**Published:** 2022-09-05

**Authors:** Adam El-Gamel, Josephenine Mak, Steve Bird, Megan N C Grainger, Gregory M Jacobson

**Affiliations:** Auckland University, Auckland, New Zealand; Science and Engineering, University of Waikato, Hamilton, New Zealand; Wellington General Hospital, Wellington, New Zealand; Science and Engineering, University of Waikato, Hamilton, New Zealand; Science and Engineering, University of Waikato, Hamilton, New Zealand; Science and Engineering, University of Waikato, Hamilton, New Zealand

**Keywords:** Ascending aortic aneurysm, Copper deficiency

## Abstract

**OBJECTIVES:**

Studies in animals have shown causal relationships between copper (Cu) deficiency and the development of thoracic aortic aneurysms (TAAs) [1, 2]. Cu deficiency is widespread in New Zealand (NZ) soils; the high soil pH from the use of lime fertilizers reduces the bioavailability of Cu for grazing animals and growing plants; this, in turn, reduces Cu availability in the NZ human food chain. Our study is a pilot study to explore associations between Cu and TAA. We measured Cu levels in aneurysmal aortic tissues in patients undergoing Bentall procedures and non-aneurysmal aortic tissue from coronary artery bypass graft patients.

**METHODS:**

Aortic samples were collected from 2 groups of patients during elective open-heart surgery over 4 months between November 2017 and February 2018. The groups were a TAA group, patients with non-syndromic aortic aneurysm and without the bicuspid aortic valve or known infectious or inflammatory condition (ANEURYSM; *n* = 13), and a control coronary artery bypass graft group (CONTROL; *n* = 44). Standardized digested dry tissue weighed samples were analysed from both groups. Tissue extraction of trace elements was carried out using HCl-H_2_O_2_ digestion and a highly sensitive analytical technique, inductively coupled plasma mass spectrometry—used to measure elemental concentrations.

**RESULTS:**

Cu concentration (mean ± SD) was significantly lower in ANEURYSM (3.34 ± 0.16 µg/g) when compared to the CONTROL group tissues (4.33 ± 0.20 µg/g) (dry weight; mean ± SD; Student's *t*-test, *P* < 0.05). Over 46% of the Aneurysm patients were Maori and live in a geographically Cu-deficient NZ territory.

**CONCLUSIONS:**

Cu deficiency may play a role in the development or progression of non-syndromic ascending aortic aneurysms in NZ. Maori patients are more at risk as they commonly live in rural NZ, dependent on locally grown nutritional sources. Further studies are required to confirm this exciting finding and to establish cause and effect relationship.

**Figure:**
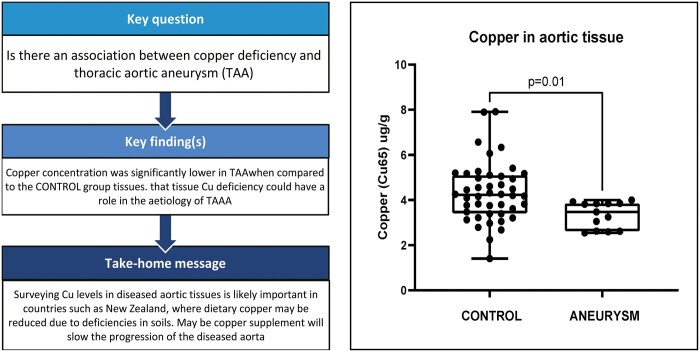

## INTRODUCTION

The pathogenesis of thoracic aortic aneurysms (TAAs) is multifactorial, with genetic and environmental factors implicated in causing the microstructural changes in content or architecture of the connective fibres elastin and collagen, which are responsible for the integrity of the aorta [3]. While relatively little research has been carried out on environmental factors in the disease, it is thought that deficiency in trace minerals may play a role, and copper (Cu) is of particular interest. Cu deficiency in animals is associated with an increased risk of aneurysm, and experimental models in animals have established a cause and effect relationship between Cu deficiency and aneurysm and dissections in pigs, mice and turkeys [4, 5].

There is significant ongoing research around the genetic factors important to thoracic ascending aortic aneurysms (TAAA) [6]. Marfan syndrome, which can arise from different mutations of the fibrillin gene, is a well-known cause of TAA and dissection. Marfan patients comprise 5–10% of TAAA cases and, without surgical intervention, have a 50% lifetime chance of developing aortic dissection [7]. Turner's syndrome and Ehlers–Danlos syndrome are also associated with an increased incidence of the aortic aneurysm. However, genetic causes for non-syndromic familial TAA have also been identified. Relevant mutations have been reported in the genes: TAA and Dissection (thought to be responsible for 20–30% of TAA), Familial Aortic Aneurysm, Transforming Growth Factor Beta Receptor, Myosin Heavy Chain, and Smooth Muscle Alpha-Actin [8]. The association of the bicuspid aortic valve with TAAA has also been noted, with the aortic valve sharing the exact embryological origin as the ascending aorta [9].

In contrast to the relative abundance of genetic study data, there are few investigations of environmental factors in the disease. It has, however, been hypothesized that deficiency in certain trace minerals may be involved in the disease aetiology—with Cu being of particular interest. In humans, the Cu-dependent enzyme lysyl oxidase (LOX) functions in cross-linking elastin and collagen fibres [10], which are key contributors to aortic stretch and strength. Cu deficiency can thus result in a disruption in aortic wall architecture and therefore cause aortic disease. Inactivation of the LOX gene using gene engineering has resulted in large ruptured aortic aneurysms in the perinatal period in mice [11, 12].

Furthermore, other animal studies have shown relationships between Cu and the risk of aneurysms. For example, in Blotchy mice—abnormal Cu absorption was related to the development of spontaneous aneurysms {Abe, 2002 #96}. In New Zealand (NZ), dairy calves and sheep grazed on Cu-deficient soils are implicated in some mineral deficiency diseases With a Cu deficiency (identified in serum levels), pigs are linked to aneurysmal aortas of nearly twice normal healthy diameter [13].

It has also been reported that Cu, Zn and chromium concentrations are significantly lower in atherosclerotic plaques sampled from abdominal aortas of deceased patients with ischaemic heart disease and acute myocardial infarction than in a control group of patients who died of accidents or other causes than atherosclerosis [14]. However, it is essential to note that this research cannot be directly extrapolated to TAA. As mentioned previously, these 2 diseases are fundamentally different, affecting different patient populations and having different embryological origins. In addition, obtaining a clear understanding is confounded by the fact that one of the most common causes of AAA is atherosclerosis, yet atherosclerosis does not always cause TAAA [7]—the 2 pathologies may, in fact, present separately.

In NZ, particularly in the country's North Island, the abundance of volcanic soils deficient in trace elements has required farming practices to include supplementation of animals and soils with trace elements including Cu and Zn to ensure the welfare of animals and plants. While it has not been fully explored, human populations living in the same environment, consuming locally produced food, may also have diets deficient in trace elements, which may have consequences on their health.

This research investigated the hypothesis that there is a significant difference in tissue levels of Cu between aneurysmal and non-aneurysmal aorta in NZ patients. The aim is to explore possible roles for Cu in the development of an aortic aneurysm.

## METHODS

### Ethics

Local district health board ethics and consent for the use of tissue for research purposes were obtained as part of standard surgical consent at this research centre. A cultural consultation process with the local Māori research review committee was also completed. The tissue is considered ‘taonga’—treasured—in Māori culture, careful steps were taken to ensure respectful handling and treatment of tissue with thought to cultural safety. The inclusion and exclusion criteria are described in Table 1. As part of the standard surgical consent process at this hospital, patients can choose whether to give consent for any removed tissue to be used for research purposes. However, for the purposes of the study, all patients were seen by the investigator as part of the recruitment process. A full explanation of the study was given to the patient and family, including the ability to withdraw consent for the use of tissue, with the return of tissue possible before time of tissue processing for analysis.

**Table 1:** Inclusion and exclusion criteria

Inclusion criteria	Exclusion criteria
ANEURYSM: Patients undergoing surgery for dilated aortic root at Waikato Cardiothoracic Unit	Genetic collagen disorders (Marfan, Ehler–Danlos, etc.) bicuspid aortic valve
CONTROL: Patients undergoing CABG who have non-aneurysmal aortas at Waikato Cardiothoracic Unit	Infective aetiology for aneurysm
Ability to give informed consent	Diagnosed inflammatory causes
Age >18 years old	Chronic post-traumatic aetiology

CABG: Coronary artery bypass graft.

### Recruitment

This prospective, observational, single-centre pilot study was conducted at a large tertiary NZ hospital (Waikato Hospital) that serves as a cardiothoracic referral centre for approximately 900 000 patients.

For the study, we have recruited 2 groups of patients over 4 months between November 2017 and February 2018. The groups were:

TAA group (ANEURYSM)—patients with aneurysmal aortas undergoing replacement (*n* = 13)

CABG control group (CONTROL)—patients undergoing CABG who have non-aneurysmal aortas (*n* = 44).

**Figure 1:**
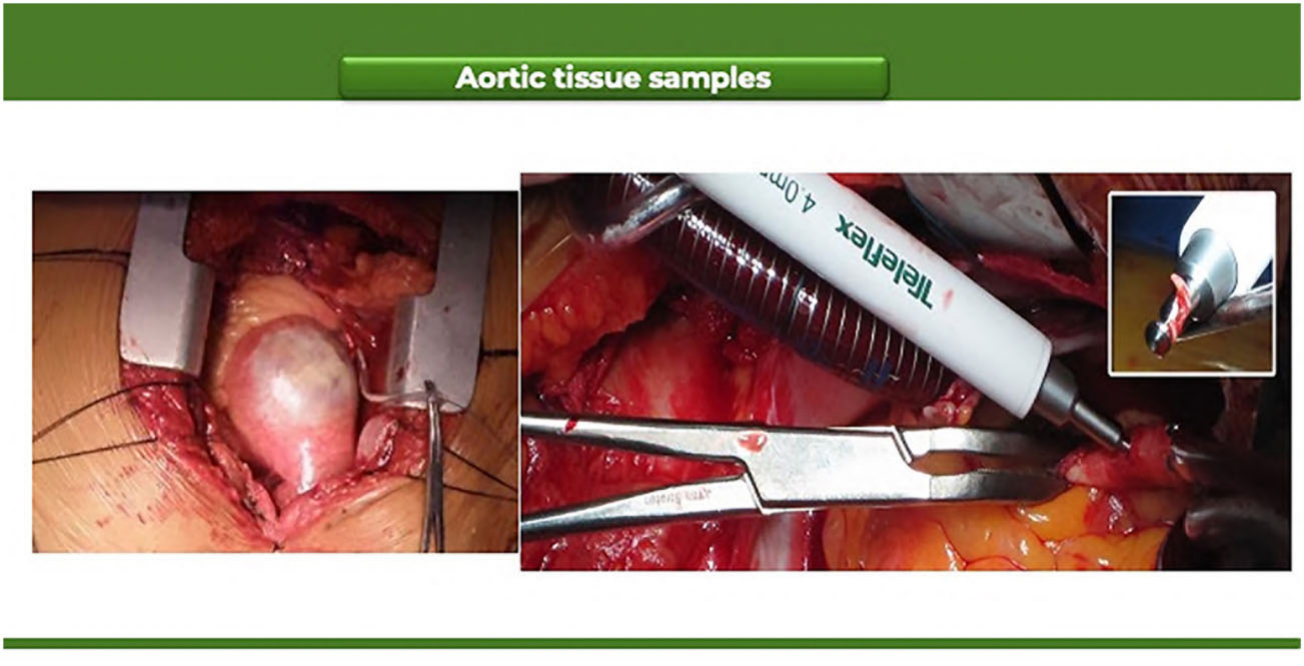
Sampling tissues during surgery.

Aneurysmal tissue was sampled from the ANEURYSM group during surgery and for the CONTROL group, the tissue was taken as an aortic 5 mm punch used to create the aortic opening during proximal anastomosis coronary artery bypass graft (CABG; Fig. 1). There were no changes to the usual operative or treatment protocol. De-identified tissue was delivered to the laboratory and stored in sterile plastic tubes at −20°C until further processing and analysis.

Measurement of Cu using inductively coupled plasma (ICP) mass spectroscopy.

CABG punches and aneurysm tissue (treated similarly to the CABG samples by punching with a 5 mm surgical punch; Myo, Pelegrina, USA) were weighed and then dried at 80°C (>3 h); with no constant weight change over 30 min considered as complete desiccation. To obtain dry weight, samples were then placed in a labelled polypropylene tube (Greiner) and reweighed using a five decimal place balance. Next, a digest solution comprising HNO_3_:H_2_O_2_:H_2_O (chemicals from Sigma-Aldrich, NZ) was prepared in a ratio of 3:2:3 and samples were digested at 80°C in a heat block for 2 h in a 10 ml volume of the solution. Method blanks are prepared in the same manner as the sample tubes. After digest, the solutions were filtered using 0.45 µM cellulose acetate filters (Minisart, Sigma-Aldrich, NZ) into 15 ml tubes and samples placed in an autoloader. Finally, the elemental composition was analysed on an ICP instrument.

Accuracy and precision were determined by calculating spike recoveries; a known amount of Cu standard (*n* = 3) was added to a set of samples; good accuracy was deemed as 100 ± 10% of the expected concentration. Recovery was acceptable for all masses analysed.

In order to control for inter-day and inter-run sample variation, ICP mass spectrometry standards of known concentration (IV-ICPMS-71A; Inorganic Ventures, USA) were run alongside study samples. Tissue masses were chosen to allow the determination of Limit of Detection, and Limit of Quantitation of the method. Blanks were run in triplicate to determine intra-sample variability.

Due to low sample masses (mean ± SD 5.47 ± 2.66) mg dry weight in the CONTROL group, the entire CABG tissue sample was used; the dry mass was recorded and estimated Cu reported as µg/g dry weight. It was usually not possible to obtain the accurate wet weight of CABG punches (CONTROL group) due to tissue storage in liquid media and rapid air drying at room temperature during preparation. In order to assess methodology sensitivity and accuracy for the detection of Cu, spikes samples were run. Sample recovery was within acceptable levels of 75–125%.

### Statistical analysis

Data distributions for Cu concentration and all recorded demographic features were tested using the Shapiro–Wilk normality test. Data were subsequently examined using Student's *T*-test (for normally distributed data), *Χ*^2^ test and Fisher's exact test (for categorical data), or the Mann–Whitney *U*-test (for non-normally distributed data). Statistical analysis was performed using GraphPad Prism 9 software (GraphPad software, Inc., La Jolla, CA, USA). For all tests, *P* < 0.05 was considered significant.

## RESULTS

### Inductively coupled plasma validation

The ICP methodology used was found to have Limit of Detection (i.e. mean Cu of blanks plus 3 times the standard deviation of blanks) of 0.61 µg/g and Limit of Quantitation (mean Cu of blanks plus 3 times the standard deviation of blanks) of 1.21 µg/g for Cu. This validates the method for measuring dry mass samples when Cu is above 1.21 µg/g, which was true of all measurements.

### Copper concentration

**Figure 2:**
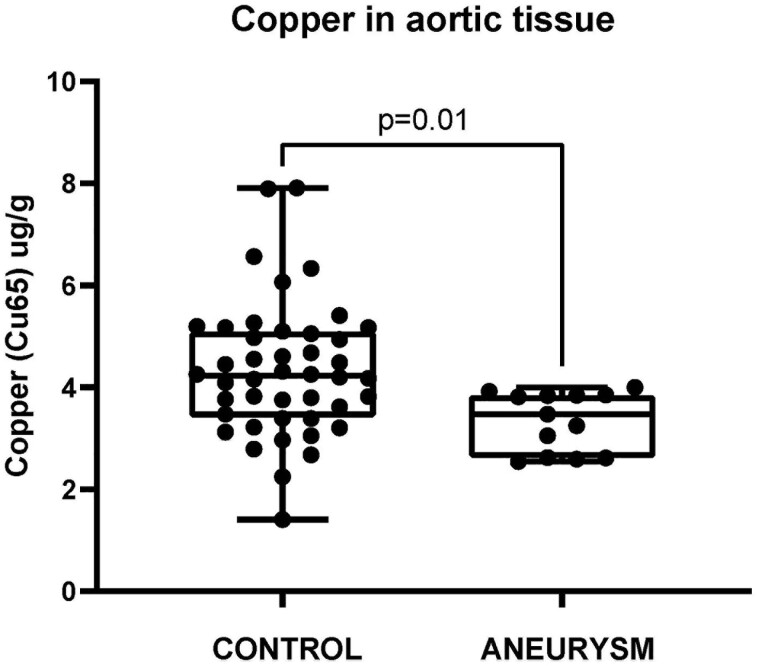
Showing Cu level for aorta tissue between CONTROL and ANEURYSM samples. *P* = 0.01 (Student's *t*-test). Cu: copper.

ICP data were obtained for 13 ANEURYSM samples and 44 CONTROL samples (all tissues had dry mass >2.5 mg). There was a significant difference in Cu level between ANEURYSM 3.34 ± 0.16 µg/g and CONTROL groups 4.33 ± 0.20 µg/g (mean ± SD; Student's *t*-test, *P* = 0.01) (Fig. 2) (Table 3).

### Baseline characteristics

From November 2017 to February 2018, 44 patients who underwent CABG and 13 patients who underwent aortic surgery for TAA or aortic dissection consented and tissue was collected for this study. Full details of patient demographics can be found in Table 2. Masses of dry tissue obtained were 5.62 ± 2.85 mg for CONTROL and 10.47 ± 0.49 mg for ANEURYSM (mean ± SD). Euroscore was the only demographic variable that differed between groups, with values of 4.82 (2.50) in CONTROL and 1.57 (1.03) in ANEURYSM groups [median % (interquartile range); *P* = 0.02]. While the study numbers were too small to allow rigorous statistical testing, we observed that Māori, who represent around 17% of the NZ population, made up 46% (6/13) of the ANEURYSM group but only 9% of the CONTROL group (4/44).

**Table 2:** Continuous variables are given as mean ± SD or median (interquartile range) and where categorical they are given as proportion (percentage)

	ANUERYSM	CONTROL	*P*-value
No. of patients	13	44	
Weight (kg)	91.4 (85.1–97.7)	87.5 (79.1–96)	0.38^a^^.^
Height (cm)	174 (169.9–178.1)	173 (169.3–176.7)	0.70^a^^.^
No. of female	2 (15%)	6 (14%)	0.87^b^^.^
BMI (kg/m^2^)	30.2 (28.2–32.2)	29.2 (26.7–31.7)	0.45^c^^.^
Age at operation (years)	64.2 (58.2–70.3)	66.9 (62.6–71.3)	0.47^a^^.^
Diabetes	1 (8%)	14 (33%)	0.03
Ethnicity			
Asian	0 (0%)	2 (5%)	
European	7 (54%)	38 (86%)	0.03
Māori	6 (46%)	4 (9%)	0.01
Euroscore II	4.82 (3.6–6)	1.57 (1.1–2.1)	0.02^c^^.^
Hypertension	13 (100%)	37 (86%)	0.15^b^
Hypercholesterolaemia	7 (54%)	36 (84%)	0.05^b^
EF (%)	58.8 (54.1–63.5)	55.7 (50.3–61.2)	0.55^c^
Obesity (BMI ≥30)	2 (22%)	20 (46%)	0.05^b^^.^
Smoking	4 (33%)	26 (61%)	0.07^b^^.^

a Student's *T*-test.

b Fisher's exact test.

c Mann–Whitney *U*-test.

nd: not determined.

**Table 3:** CABG versus aneurysmal tissue copper level

Tissue group	*N*	Copper µg/g (mean ± SD)	95% CI	*P*-value
CONTROL	44	4.33 ± 0.20	3.94–4.73	*0.01*
ANEURYSM	13	3.34 ± 0.16	2.98–3.69	

*P*-value from Student's *t*-test.

CABG: Coronary artery bypass graft.

## DISCUSSION

Thoracic ascending aortic aneurysms (TAAA) are usually asymptomatic. This disease is often discovered incidentally by imaging via computed tomography, magnetic resonance imaging or echocardiography, or not detected until some catastrophic medical event [8]. As aneurysms are generally asymptomatic, it is difficult to determine their epidemiology. Besides, dissections are occasionally misdiagnosed, e.g. as myocardial infarction. TAAA appears to be increasing in frequency; a trend was seen in the USA, Scotland, the Netherlands, and England and Wales [15]. However, it is difficult to know if this observed trend represents an actual increase in the proliferation of the disease or if it is due to improved diagnostic methodology.

The aortic aneurysm has multifactorial pathogenesis, with contributing genetic and environmental factors interacting to variable degrees leading to the degradation of aortic wall components [15].

Although the aortic aneurysm macroscopic and histological characteristics are well-established, the dynamic biological process that produces these changes is exceptionally complicated and incompletely determined.

Because of the absence of both necrosis or cysts in the histopathology examination of the wall, the term cystic medial necrosis describing pathological changes in the TAA is considered as an inaccurate term [16]. The main finding is the degeneration of elastin accumulation of proteoglycans [16].

The primary ingredient of the aortic wall is the extracellular matrix; the extracellular matrix constitutes more than half of the wall mass and contains mainly collagens and elastin.

Recent studies proposed that the risk of developing AAA is due to interaction between multiple genetic loci and on the exposure of patients to environmental or other complex risk factors, such as nicotine, diet and other health behaviours. We have reported the increased prevalence of acute aortic syndrome in NZ north island [17], and we have accumulated knowledge from the NZ farming industry relating to Cu deficiency impact on animal and plant life. It is reasonable to look for Cu as an extra risk factor that may contribute to the development or progression of TAAs in the NZ population.

Subclinical chronic Cu deficiency may be a relevant nutritional problem in humans which could be exacerbated by interaction with other trace elements competition, particularly Zn, which is also commonly used as a soil and animal supplement in NZ.

Disruption of elastin has been particularly implicated in AAA development and disease progression, while collagen degradation is thought to be more critical in AAA rupture.

Normally, elastogenesis is confined predominantly to foetal life and infancy, and matured elastin last for the whole lifetime. Elastin half-life is about 40 years [18]. Age and disease degrade and fragment elastic fibres, leading to increased stiffness of the arterial wall [18]. The vascular wall reaction to increased mechanical stress stimulates vascular cells (Smooth muscle cells (SMCs), Endothilial cells (ECs) and fibroblasts) to make elastin and tropoelastin, but these tropoelastins fail to cross-link into elastic fibres [18].

Because tropoelastin is subjected to oxidative deamination or cross-linking by LOX. We hypothesized that the deficiency of Cu might diminish the Cu-dependent enzyme LOX activity, especially in the early phase of infancy [19].

Elastin degradation is one of the most significant signs of human blood vessel aneurysms. Serum elastin peptides were reported to be significantly elevated in AAA patients. Aortic tissue extract HPLC analysis confirmed the reduction of elastin cross-links in human AAAs [20].

When ageing or tissue injury damages elastin, elastic fibres are mainly not replenished because elastin gene expression is switched off in adults. In its place, collagens are generated to replace the lost elastin fibres, stiffening the arterial wall [19]. Collagen and elastin cross-linking provide structural cohesion of the arterial wall. LOX promotes cross-link formation in nascent fibrils of collagen and elastin [1].

So if cross-linking enzymes are less active, it could be because of Cu deficiency or genetic signal. However, AAA and aneurysms of Marfan syndrome show altered collagen architectures with loss of collagen knitting. This may be due to the type of collagens and the methods applied. [21]

Ceruloplasmin carries cu in the plasma, and low plasma or serum levels do not correlate with tissue levels. Cu is assimilated into the ceruloplasmin composition by metallothionein during synthesis in the liver. The absence of metallothionein (Wilson's disease) can result in the decrease of serum ceruloplasmin levels, leading to the accumulation of Cu in the liver, kidneys, skin, brain (with associated neurological symptoms) [22]. Ceruloplasmin level may be high due to infection, inflammation or oestrogen influence, and diabetes is also associated with raised plasma Cu concentration. Several factors not directly related to the Cu status, but often related to changes in ceruloplasmin levels, make plasma Cu an unreliable marker of Cu status, except in more severe deficiency states; for these reasons, we elected to test Cu levels in tissues.

Cu levels in TAA have not been well-explored. One study reported lower Cu in aortic tissues in TAA (*n* = 18) compared to a control (autopsy) group (*n* = 12) (mean ± SD: TAA 0.9 ± 0.2 μg/g vs controls 1.3 ± 0.4 μg/g wet tissue weight; *P* < 0.01) [23]. Inflammatory aneurysms have a high load of leucocytes, which have significant levels of Cu [24].

The problem can also be exaggerated with other trace elements interference.

Large doses of Zn can trap Cu in the intestinal mucosal cells bound to metallothionein until the mucosal cells are shed. The quantity of Zn required is well above the healthy intake, but the margin seems to be narrower in humans, especially in premature infants. This mechanism is exploited in the Zn therapy of Wilson disease, in which the dose required (50 mg before each meal) is ∼5 times the nutritional requirement. Zinc administered for other purposes can cause Cu deficiency [25].

Our data indicate that Cu is significantly reduced (∼25% less) in aorta tissue from aneurysmal versus non-aneurysmal patients. In some studies, these levels are similar to those reported previously for aortic wall Cu concentrations.

Cu may protect against coronary artery disease [26] and previous findings showed that Cu concentration is lower in abdominal aortas from patients with atherosclerotic disease. So, the choice of CABG patients as a control group, where Cu levels may be lower in the thoracic aorta when compared to healthy patients, may, in fact, underestimate the scale of the difference in Cu level seen in this study.

Surveying Cu levels in diseased aortic tissues is likely important in countries such as NZ, where dietary Cu may be reduced due to deficiencies in soils. Trace element levels, including Cu, within NZ soils, vary greatly, with widespread Cu deficiency reported. Patient geographical histories may allow a better understanding of roles of Cu deficiency in TAAA and historical Cu deficient is relevant as, while collagen has a relatively short turnover, the half-life of elastin is measured in tens of years [18].

## CONCLUSION

This study highlights that tissue Cu deficiency could have a role in the aetiology of TAAA. It is hoped that this study contributes towards the growing body of knowledge around the pathogenesis of TAAA, and ultimately helps improve clinical outcomes.

### Future directions

This exploratory study sets the basis for a larger prospective trial confirming the observation of lower Cu levels in aneurysmal tissue. Control tissues from cadavers with the normal aorta and no CAD would be useful for subsequent studies; studying the activity of LOX at the same setting would establish the mechanisms that connect both hypotheses.

## Data Availability

All relevant data are within the manuscript and its Supporting Information files.

## Notes

The data underlying this article will be shared on reasonable request to the corresponding author.

## ABBREVIATIONS

CABG: Coronary artery bypass graft
Cu: Copper
ICP: Inductively coupled plasma
LOX: Lysyl oxidase
TAA: Thoracic aortic aneurysms
TAAA: Thoracic ascending aortic aneurysms
NZ: New Zealand
